# The role of leaf superoxide dismutase and proline on intra-specific photosynthesis recovery of *Schima superba* following drought

**DOI:** 10.1038/s41598-024-59467-9

**Published:** 2024-04-17

**Authors:** Honglang Duan, Changchang Shao, Nan Zhao, Defu Wang, Víctor Resco de Dios, David T. Tissue

**Affiliations:** 1 Institute for Forest Resources & Environment of Guizhou, College of Forestry, Guizhou University, Guiyang, 550025 China; 2 Jiangxi Provincial Key Laboratory for Restoration of Degraded Ecosystems & Watershed Ecohydrology, Nanchang Institute of Technology, Nanchang, 330099 China; 3 Research Center of Sichuan Old Revolutionary Areas Development, Sichuan University of Arts and Science, Dazhou, 635000 China; 4 Department of Crop and Forest Sciences, University of Lleida, 25198 Lleida, Spain; 5 Hawkesbury Institute for the Environment, Hawkesbury Campus, Western Sydney University, Richmond, NSW 2753 Australia; 6 Global Centre for Land-Based Innovation, Hawkesbury Campus, Western Sydney University, Richmond, NSW 2753 Australia

**Keywords:** Gas exchange, Water status, Biochemistry, Extreme drought, Recovery, Intraspecies, Ecophysiology, Ecology, Plant sciences

## Abstract

Understanding the physiological and biochemical responses of tree seedlings under extreme drought stress, along with recovery during rewatering, and potential intra-species differences, will allow us to more accurately predict forest responses under future climate change. Here, we selected seedlings from four provenances (AH (Anhui), JX (Jiangxi), HN (Hunan) and GX (Guangxi)) of *Schima superba* and carried out a simulated drought-rewatering experiment in a field-based rain-out shelter. Seedlings were progressively dried until they reached 50% and 88% loss of xylem hydraulic conductivity (PLC) (i.e. *P*_50_ and *P*_88_), respectively, before they were rehydrated and maintained at field capacity for 30 days. Leaf photosynthesis (*A*_sat_), water status, activity of superoxide dismutase (SOD), and proline (Pro) concentration were monitored and their associations were determined. Increasing drought significantly reduced *A*_sat_, relative water content (RWC) and SOD activity in all provenances, and Pro concentration was increased to improve water retention; all four provenances exhibited similar response patterns, associated with similar leaf ultrastructure at pre-drought. Upon rewatering, physiological and biochemical traits were restored to well-watered control values in *P*_50_-stressed seedlings. In *P*_88_-stressed seedlings, Pro was restored to control values, while SOD was not fully recovered. The recovery pattern differed partially among provenances. There was a progression of recovery following watering, with RWC firstly recovered, followed by SOD and Pro, and then *A*_sat_, but with significant associations among these traits. Collectively, the intra-specific differences of *S. superba* seedlings in recovery of physiology and biochemistry following rewatering highlight the need to consider variations within a given tree species coping with future more frequent drought stress.

## Introduction

Drought has negative impacts on plant growth and development^1^. In recent decades, increasing drought-induced tree mortality in natural forests have been observed worldwide^2–4^, which may affect forest composition and functioning. The frequency and duration of droughts are also predicted to increase with climate change in the future, thus leading to a greater risk of drought-induced tree mortality^5,6^. Tree responses to drought are coordinated by different types of physiological functions, such as carbon and water relations and biochemical adjustments^7–10^. Therefore, enhanced understandings of the coordination among physiological functions during drought as well as recovery and the potential variation in a given species, will help to determine the physiological plasticity of tree species under drought stress and predict tree species physiological performance in the context of climate change.

Drought stress affects carbon and water relations^11,12^. For instance, growth, photosynthesis and foliar respiration are often decreased by drought, depending on the severity of drought stress. Leaf water potential and relative water content (RWC) exhibit declines under drought, mainly determined by the interplay between stomatal regulation and xylem hydraulic adjustments^13–15^. Drought stress also induces biochemical changes, such as increases in proline (Pro) concentration for osmotic regulation and in antioxidant enzymes for reducing membrane damage caused by lipid peroxidation, under mild and moderate drought. However, it is currently considered that this protective capacity is limited because the activities of superoxide dismutase (SOD; an enzyme that detoxifies toxic singlet oxygen) and peroxidase (an enzyme that scavenges H_2_O_2_) have been reported to decline under severe drought^16–20^. Nevertheless, the coordination between carbon and water relations with biochemical adjustments during extreme drought has seldom been quantified.

Intra-specific variation is a factor introducing further uncertainty in tree physiology and biochemistry in response to drought. For instance, variation in RWC, photosynthetic rate, water use efficiency and SOD activity of droughted *Camptotheca acuminata* seedlings was related with inter-provenance differences in drought tolerance^21^*.* Similarly, *Balanites aegyptiaca* showed intra-specific differences in drought sensitivity associated with different contents of tocopherol and antioxidant enzymes^18^. In addition to physiology and biochemistry, leaf structure, such as chloroplast ultrastructure, may also impact drought responses within tree species. For example, the length, width and width/length of chloroplasts were higher in drought-resistant varieties of sour orange (Kliaa) and sugarcane (F172)^22,23^. Previous studies on tree drought responses have examined carbon and water relations, biochemical and ultrastructural responses independently or jointly, but it remains unclear how these processes were coordinated during drought or if the coordination differs among provenances.

After the dry spell is broken by soil rewatering, different degrees of recovery in growth and physiology have been observed among tree species and provenances, usually accompanied by changes in biochemistry^18,24^. For example, *Castanopsis chinensis* exhibited a full and rapid recovery of photosynthesis after rewatering, but *Schima superba* and *Syzygium rehderianum* took more time to recover from drought leading to an approximate 50% loss of hydraulic conductivity (PLC)^25^. Additionally, the photosynthetic recovery of *Fagus sylvatica* from a xeric provenance was faster than from a mesic provenance^26^. Post-drought recovery depends on the degree of antecedent drought stress^27–29^. The recovery of most physiological parameters is relatively rapid after mild stress, while more severe stress may lead to cell damage and loss of biochemical function^12,20,30,31^.

To sum up, few studies have examined the recovery of leaf biochemistry after a very intense drought and the links between biochemical and physiological recovery remain unclear. Studies regarding the capacity of recovery from extreme drought stress are less common in jointly addressing gas exchange, water relations and biochemical traits (e.g. traits related to osmotic adjustment and cell membrane protection), particularly in assessing intra-specific variation of tree species. It remains unclear to what extent leaf gas exchange, water relations and biochemistry can recover from extreme drought stress, and whether the coordination of these physiological and biochemical traits (if any) would vary among populations within a given tree species. Accordingly, further in-depth study of tree physiological and biochemical responses to extreme soil drought and post-drought rewatering in terms of intra-specific variation, will allow us to more accurately predict forest responses to variable rainfall patterns under climate change.

**Figure 1:**
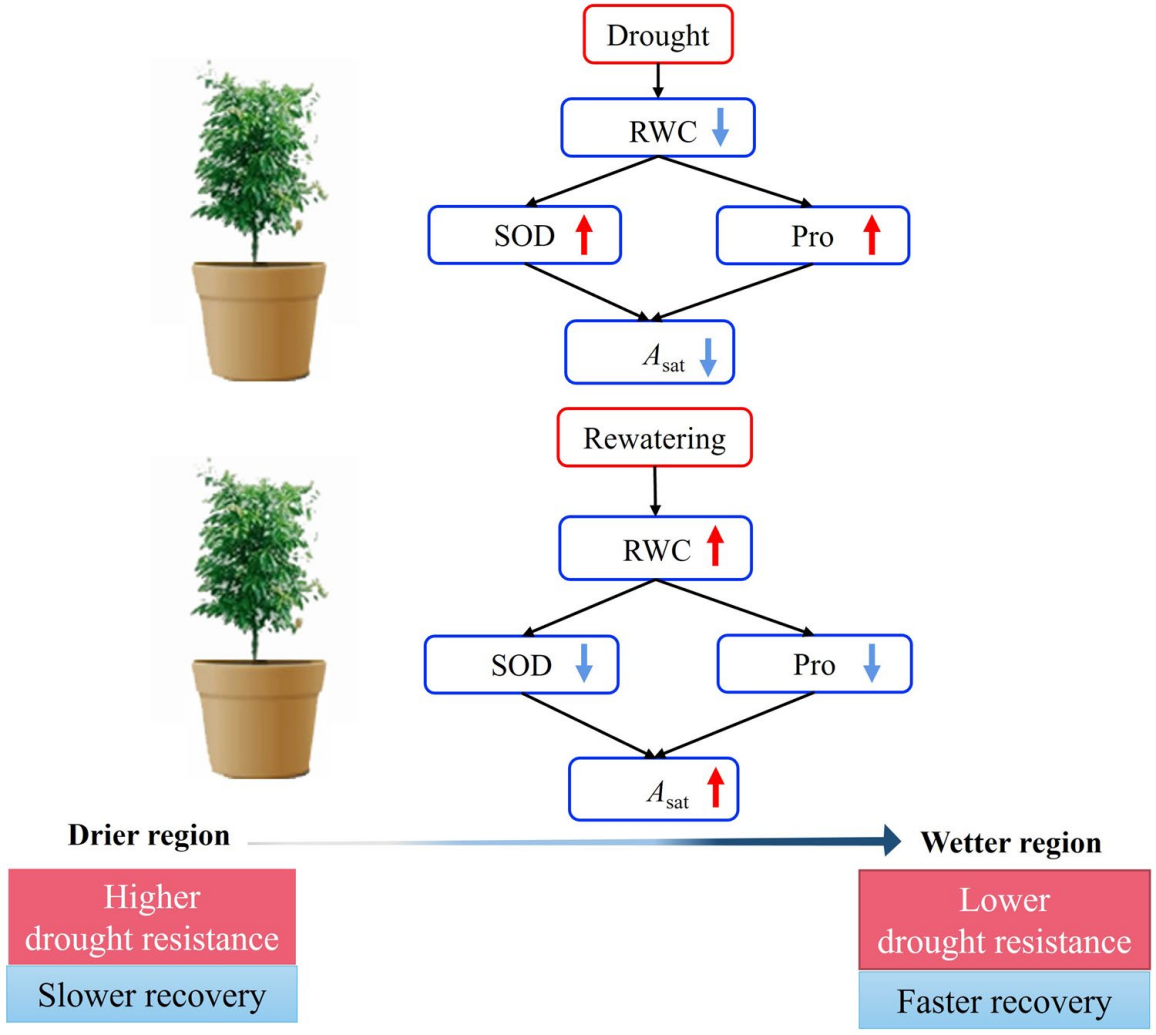
Hypothesized relationships of physiological and biochemical traits of *S. superba* seedlings during drought and rewatering in this study. We hypothesized that provenances from drier region would exhibit higher drought resistance than provenances from wetter region, accompanied with slower reductions in RWC, and photosynthesis but higher biochemical adjustment. Upon rewatering, these parameters had slower recovery in provenances from drier areas. The red arrows indicated rise and blue arrows represent decline.

Here, *S. superba* was selected as the study species, an evergreen broad-leaved tree species widely distributed across subtropical China with important ecological and ornamental values^32,33^ that is likely to be affected by increasing drought stress in the coming decades^34^. Previous research has demonstrated the effects of drought and recovery on photosynthetic, hydraulic and biochemical responses of *S. superba* seedlings^25,35–38^. However, it remains to more clearly explore its intra-specific variation of physiological and biochemical responses during drought and recovery and the links among traits. In the current study, the dynamics of Pro concentration and SOD activity were assessed at different degrees of drought stress (i.e. *P*_50_ and *P*_88_), which represent varying levels of PLC (i.e. around 50% and 88%), respectively, and during the 30 d post-drought recovery from each drought level. During recovery, correlations between gas exchange, water relations and biochemical responses were determined. The leaf water and ultrastructure traits among provenances was also examined at pre-drought conditions, which can provide further information on intra-specific difference in leaf structure, water use strategy and drought tolerance, enhancing the understanding of photosynthetic responses during drought and recovery. The present study aimed to determine whether leaf carbon–water physiology and biochemistry of *S. superba* seedlings were coordinated as they approached extreme drought stress and during rewatering, and whether intra-specific differences exist. More specifically, this research sought to answer the following questions: (i) how do biochemical responses change among provenances as drought stress is intensified, as well as during subsequent recovery? (ii) Are biochemical responses associated with changes in photosynthesis and water status? If any, are associations different among provenances? Regarding physiological and biochemical responses, it is hypothesized that provenances from drier region would exhibit higher drought resistance than provenances from wetter region, accompanied with slower reductions in RWC and photosynthesis but higher biochemical adjustment. Upon rewatering, these parameters had slower recovery in provenances from drier areas (Fig. 1).

## Methods

### Plant material and experimental design

Seeds of *S. superba* were collected from four locations along gradients of MAP (1124–1796 mm; increasing from AH, JX, HN to GX) and MAT (16.4–22.4 °C; increasing from AH, HN to GX) across subtropical China (see detailed provenance information in the earlier study^9^). In March 2019, potted seedlings (1-year-old) generated from seeds (7.6 l) were grown in a rain-out shelter (with 15% of natural sunlight reduced by the PVC shelter). Each pot contained about 6 kg of air-dried red soil (Quaternary Red Earth) with one seedling planted. Thirty plants from each provenance were used for this experiment.

In August 2019, seedlings were randomly assigned to each of the two watering treatments: well-watered treatment (n = 10 per provenance) and drought treatment (n = 10 per provenance per PLC level). The soil water content in the well-watered treatment was maintained at field capacity throughout the experiment. By contrast, seedlings in the drought treatment received no water until the xylem water potential reached each of the target levels for a given provenance (i.e. *P*_50_ and *P*_88_), which occurred, on average, at − 2.4 MPa and − 3.7 MPa, respectively. Details about determining *P*_50_ and *P*_88_ can be found in the previous study^9^. Once the target PLC was achieved, a subset of seedlings (n = 6–7) at each PLC level was allowed to recover by rewatering the soil to field capacity. The recovery process lasted 30 days and post-drought measurements and samplings were conducted regularly (0, 7, 15, 30 days) during the recovery period.

### Minimum leaf conductance (g_min_) and carbon isotopic measurements

According to the method of Sack and Scoffoni^39^, recent fully expanded leaves from each provenance were sampled at pre-drought, and then, minimum leaf conductance (*g*_min_; the rate of water loss through the leaf when stomata are closed) was estimated. Leaf area was measured using a Licor-3100A (Li-Cor Inc., Lincoln, NE, USA). Samples were then dried in a growth chamber, with the air temperature of 25 °C, and a light intensity of < 5 μmol m^−2^ s^−1^. Afterwards, samples were weighed every 20 min at 6 to 15 intervals using a high precision balance. The *g*_min_ (mmol m^−2^ s^−1^) was calculated from the slope of the linear part of leaf mass vs. time regression in conjunction with chamber VPD and leaf area^40^.

Other leaf samples were placed in the oven at 110 ℃ for 1 h and then oven-dried at 70 ℃ for at least 72 h. Leaf carbon isotopic composition (δ^13^C, ‰) was measured on these dried samples, using a PE2400 elemental analyzer (PerkinElmer, USA) connected to an IsoPrime100 isotope ratio mass spectrometer (Elementar, Germany).

### Leaf turgor loss point (Ψ_tlp_) determination

Pressure–volume (P–V) curves were conducted on one leaf from each of five to seven seedlings per provenance at pre-drought, according to the bench drying method following the protocol from Prometheus (by Lawren Sack, Jessica Pasquet-Kok and Megan Bartlett). Leaf water potential and relative water content were monitored periodically over the bench dehydration. Leaf water potential was measured with PMS-Model 1505D Scholander-type pressure chamber (PMS instruments, Corvalis, Oregon USA). The turgor loss point (*Ψ*_tlp_) was then estimated from P–V curves.

### Leaf photosynthetic measurements

Leaf photosynthetic measurements (photosynthesis under saturating light; *A*_sat_, µmol m^−2^ s^−1^) were taken on recent, fully expanded leaves of four replicates in the morning by Licor-6400 (Li-Cor, Lincoln, NE, USA) equipped with a red‐blue light source (6400-2B). The conditions inside the chamber were set as photosynthetic photon flux density (PPFD) of 1500 µmol m^−2^ s^−1^, [CO_2_] of 400 μmol mol^−1^, mid-day air temperature (31 ℃) and leaf-to-air VPD of 2.1 kPa. The RH ranged between 60 and 80% across provenances over the experimental period. Measurements were then made after reaching steady-state conditions (5–10 min).

### Leaf relative water content (RWC) measurements

Leaves (four seedlings per treatment from each provenance) were sampled during drought and rewatering stages. Leaf fresh weight (*W*_f_) was determined immediately after sampling and turgid weight (*W*_t_) was measured after 12 h rewatering in the water. Dry weight (*W*_d_) was determined after 72 h oven-drying at 70 °C and RWC (%) was calculated as:

1 $${\text{RWC}}=\frac{{W}_{{\text{f}}}-{W}_{{\text{d}}}}{{W}_{{\text{t}}}-{W}_{{\text{d}}}}\times 100.$$

### Superoxide dismutase (SOD) and proline (Pro) assays

About 0.2 g liquid nitrogen frozen leaf sample (four replicate seedlings per treatment from each provenance) was extracted with 3 ml phosphate buffer and then grinded. The mixture was then centrifuged at 10,000 rpm at 4 °C for 15 min and the nitrobluetetrazolium method was applied to the supernatant to determine SOD activity^41^. About 0.2 g of liquid nitrogen frozen leaf sample was placed into a 10 ml glass tube, and 5 ml (3%; v/v) sulfosalicylic acid solution was added to each glass tube. After extraction in boiling water for 10 min, the cooled filtrate of leaf sample was then used to determine the proline (Pro) concentration using guaiacol colorimetric method^41^.

### Leaf ultrastructure determination

Recent fully expanded leaves from three seedlings per provenance were selected randomly prior to drought treatment. About 1 mm^3^ size samples were cut in the Transmission Electron Microscope (TEM) fixative (G1102, Servicebio) and then were transferred into an EP tube with TEM fixative for further fixation along with vacuum extraction. Samples were then fixed at room temperature for 2 h and then at 4 °C for preservation. Subsequently, samples were fixed with 1% O_s_O_4_ in 0.1 M phosphate buffer (PB) (pH 7.4) for 7 h at room temperature and were rinsed in 0.1 M PB (pH 7.4). Samples were then dehydrated with a series of ethanol concentration (30%, 50%, 70%, 80%, 95%, 100%, 100%, for 1 h each) and different mixtures of ethanol and acetone (3:1, 1:1 and 1:3, for 0.5 h each; 1:0 for 1 h), and were finally embedded in epoxy resin (Epon 812). Additionally, resin blocks were cut to 60–80 nm thin on the Ultra microtome (UC7, Leica, German) before staining (2% uranium acetate alcohol solution avoid-light staining for 8 min, rinsed in 70% ethanol for 3 times and then in ultra pure water for 3 times; 2.6% Lead citrate avoid-CO_2_ staining for 8 min, and then with ultra pure water for 3 times). Images were taken with a TEM (HT7800/HT7700, Hitachi, Japan) and the relevant parameters were quantified using Image-ProPlu 6.0 (Media Cybernetics, Inc., Rockville, MD, USA).

### Data analysis

Data analysis was performed in SPSS 18.0 (SPSS, Chicago, USA). Pre-drought traits were analyzed among provenances using one-way analysis of variance (ANOVA) followed by Tukey post hoc tests. At each time point along the experimental period, two-way ANOVA was used to analyze the effects of PLC levels and provenances on SOD and Pro, followed by one-way ANOVA and Tukey post hoc tests. The homoscedasticity and normality were checked prior to analyses and results were considered significant at *P* < 0.05. Furthermore, the relationships among traits were assessed and fitted using linear functions where possible.

### Relevant legislations, permitting and consent

The seeds were collected under the permission of the forest owners.

## Results

### Leaf physiological and structural traits at pre-drought

**Figure 2:**
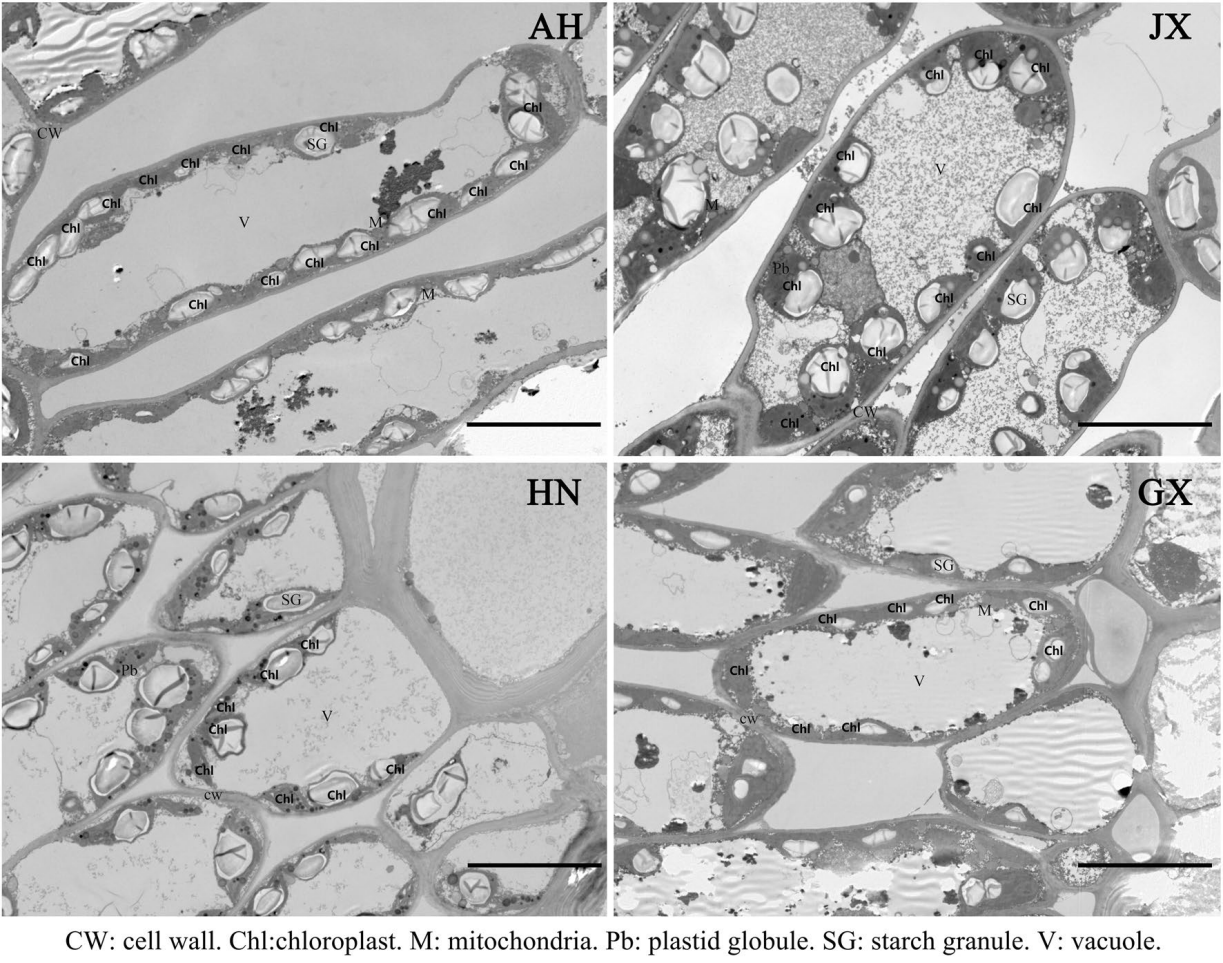
Leaf ultrastructural traits for all provenances of *S. superba* seedlings at pre-drought. AH, JX, HN, GX Anhui, Jiangxi, Hunan and Guangxi, respectively. *CW* cell wall, *Chl* chloroplast, *M* mitochondrion, *Pb* plastid globule, *SG* starch grain, *V* vacuole. The horizontal bars indicate the scale (10 μm).

At pre-drought, *Ψ*_tlp_ did not differ significantly among provenances (Table 1). The ^13^C value was higher in GX provenance than other provenances, while *g*_min_ was higher in HN provenance. In addition, there were no significant differences in leaf ultrastructural traits among provenances (Table 2, Fig. 2), showing that leaf ultrastructure may have minimal role in determining drought responses of *S. superba* seedlings in this short-term drought study.

**Table 1:** Leaf water characteristics of *S. superba* seedlings from four provenances at pre-drought.

Provenances	*Ψ*_tlp_ (MPa)	δ^13^C (‰)	*g*_min_ (mmol m^−2^ s^−1^)
AH	− 1.8 (0.1) a	− 29.1 (0.3) ab	2.5 (0.3) b
JX	− 2.1 (0.1) a	− 30.1 (0.2) b	1.9 (0.2) b
HN	− 1.9 (0.1) a	− 29.6 (0.2) b	4.4 (0.4) a
GX	− 2.2 (0.1) a	− 27.9 (0.5) a	2.8 (0.4) b

Among them: AH, JX, HN, GX represents Anhui, Jiangxi, Hunan and Guangxi, respectively. *Ψ*_tlp_ denotes turgor loss point (n = 5–7). δ^13^C represents ^13^C isotopic composition (n = 3). *g*_min_ denotes leaf minimal stomatal conductance (n = 5–6). The values in the table represent the means and SE.

Different letters denote significant differences among provenances at pre-drought conditions (*P* < 0.05).

**Table 2:** Leaf ultrastructural traits of *S. superba* seedlings from the four provenances.

Provenances	AH	JX	HN	GX
Chloroplast number	7.1 (2.9) a	10.0 (1.2) a	6.4 (0.9) a	8.4 (2.4) a
Chloroplast length (μm)	6.4 (0.3) a	6.4 (0.4) a	6.2 (0.4) a	6.2 (0.2) a
Chloroplast width (μm)	2.5 (0.1) a	3.0 (0.2) a	2.9 (0.2) a	2.6 (0.3) a
Chloroplast area (μm^2^)	11.9 (1.0) a	14.5 (1.0) a	14.0 (1.7) a	12.4 (1.6) a
Starch granule	1.8 (0.4) a	1.2 (0.1) a	1.8 (0.2) a	1.8 (0.4) a
Starch granule length (μm)	2.5 (0.2) a	3.5 (0.2) a	3.1 (0.4) a	3.3 (0.7) a
Starch granule width (μm)	1.2 (0.1) a	1.8 (0.2) a	1.7 (0.3) a	1.7 (0.3) a
Starch granule area (μm^2^)	2.2 (0.4) a	5.2 (0.6) a	4.5 (1.2) a	5.0 (2.1) a

Values are means ± SE (n = 3).

Different letters denote significant differences among provenances at pre-drought conditions (*P* < 0.05).

### Leaf physiological and biochemical responses during drought and post-drought recovery

In our earlier company study^9^, temporal dynamics of RWC and relative *A*_sat_ were presented, showing similar patterns of drought responses but divergent recovery of relative *A*_sat_ across provenances (faster recovery in HN), with RWC (3 days) recovered earlier than relative *A*_sat_ (≥ 7 days). Here, this study further analyzed correlations of RWC and original *A*_sat_ with SOD activity and Pro concentration, which were not presented previously.

**Figure 3:**
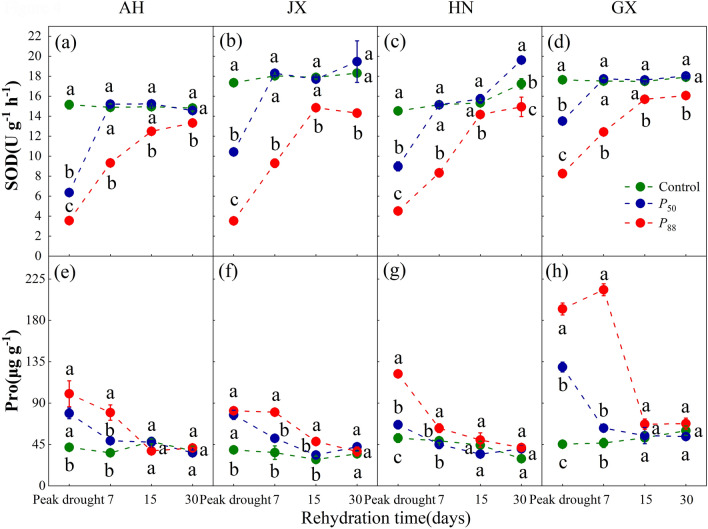
Superoxide dismutase (SOD) activity and proline (Pro) concentration for all provenances of *S. superba* seedlings at target PLC levels and following rewatering. Values are means ± SE (n = 4). Different letters denote significant differences among PLC levels at a given sampling date (*P* < 0.05).

PLC levels (i.e. different degrees of drought) had significant effects on SOD and Pro (*P* < 0.0001 for both cases), with significant interactive effects with provenance (Two-way ANOVA: *P* < 0.0001 for all time points). SOD activity decreased and Pro rose as PLC increased. In addition, SOD activity returned to control values in *P*_50_ seedlings after 7 days of rewatering. By contrast, it remained lower than that of controls in *P*_88_ seedlings (16.5%, 17.0%, 7.6%, 10.3% for AH, JX, HN, GX after 15 days of rewatering, respectively), indicating that HN provenance had relatively faster recovery (Fig. 3a–d). Pro increased with the degree of drought stress (Fig. 3e–h). Pro concentration returned to control values in *P*_50_ seedlings after 7 days of rewatering. However, under *P*_88_, Pro in AH, HN and GX seedlings took 7–15 days to recover to control levels, while those in JX seedlings recovered after 15 days.

**Figure 4:**
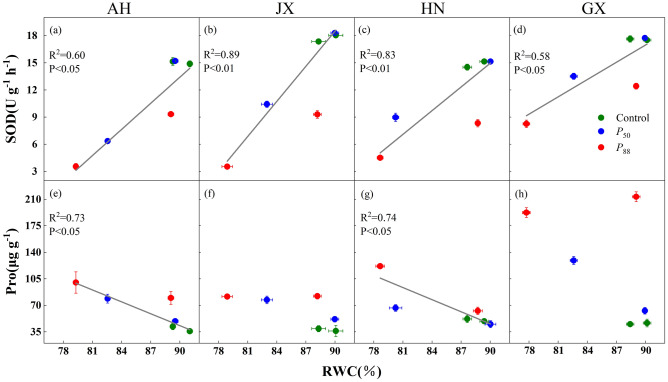
Relationship between superoxide dismutase (SOD) activity and leaf relative water content (RWC) (**a–d**), proline (Pro) concentration and RWC (**e–h**) for four provenances of *S. superba* seedlings. Values are means ± SE (n = 4). Fitted equations are as follows: (**a**) SOD = 1.0RWC − 73.1 (slope 95% CI 0.3, 1.6); (**b**) SOD = 1.3RWC − 97.9 (95% CI 0.9, 1.7); (**c**) SOD = 0.9RWC − 63.6 (95% CI 0.5, 1.2); (**d**) SOD = 0.6RWC − 40.3 (slope 95% CI 0.2, 1.1); (**e**) Pro =  − 5.2RWC + 514.0 (slope 95% CI − 7.9, − 2.5); (**g**) Pro =  − 5.3RWC + 524.8 (slope 95% CI − 8.0, − 2.6). The data used was sampled from peak drought and 7 days after rewatering.

**Figure 5:**
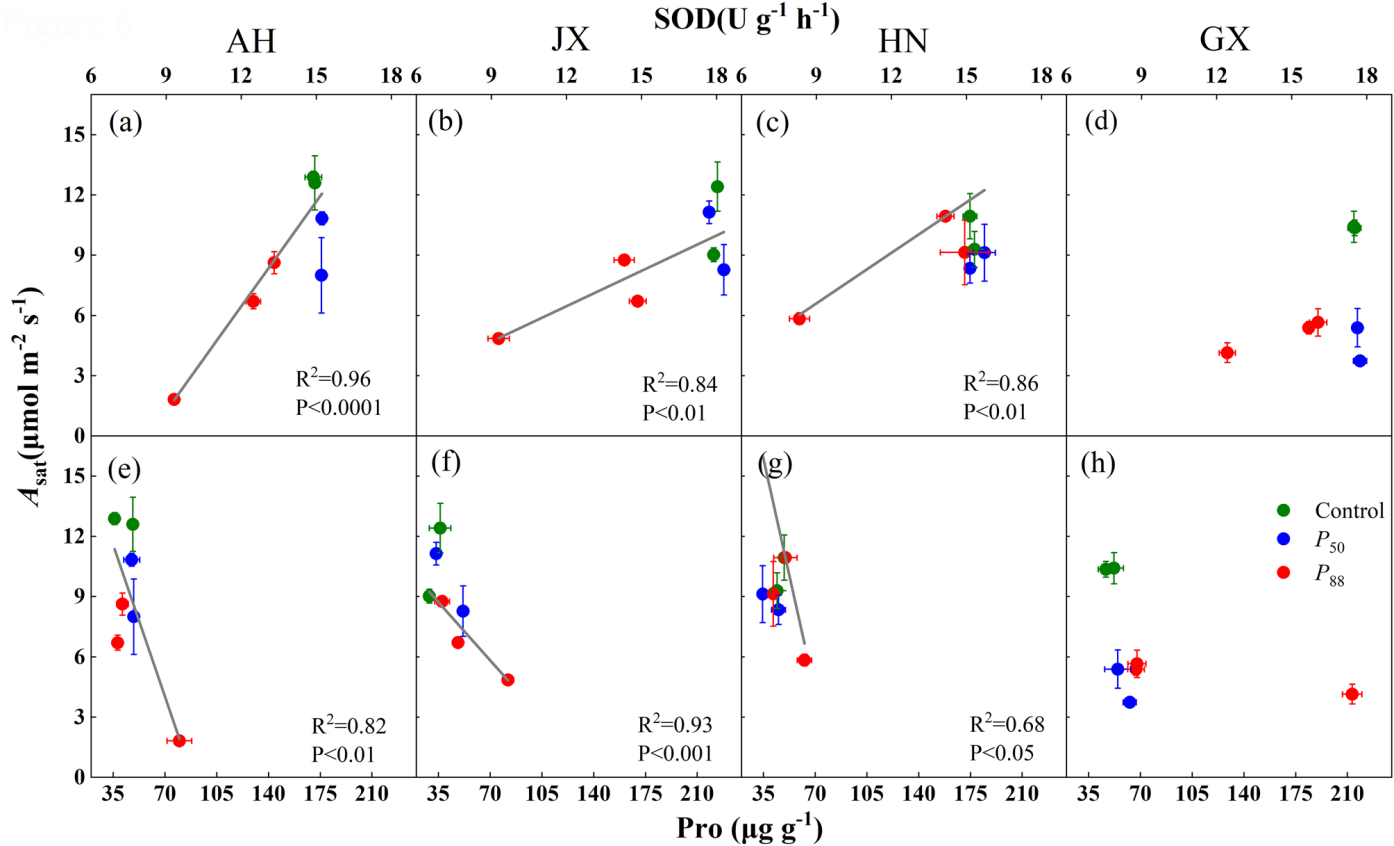
Relationship between leaf photosynthesis under saturating light (*A*_sat_) and superoxide dismutase (SOD) activity (**a–d**), *A*_sat_ and proline (Pro) concentration (**e–h**) for four provenances of *S. superba* seedlings. Values are Means ± SE (n = 4). Fitted equations are as follows: (**a**) *A*_sat_ = 1.7SOD − 14.4 (slope 95% CI 1.47, 2.01); (**b**) *A*_sat_ = 0.6SOD − 0.6 (slope 95% CI 0.39, 0.79); (**c**) *A*_sat_ = 0.8SOD − 1.0 (slope 95% CI 0.57, 1.11); (**e**) *A*_sat_ =  − 0.2Pro + 19.1 (slope 95% CI − 0.3, − 0.14); (**f**) *A*_sat_ =  − 0.1Pro + 11.6 (slope 95% CI − 1, − 0.06); (**g**) *A*_sat_ =  − 0.3Pro + 27.5 (slope 95% CI − 0.51, − 0.15). The data used was sampled from peak drought, 7 days and 15 days (for *P*_88_ only) after rewatering.

The recovery of SOD and Pro in seedlings following rewatering depended largely on the degree of drought stress and provenances (Fig. 3). Their recovery in all provenances exposed to *P*_50_-stress was more rapid than seedlings exposed to *P*_88_-stress. SOD exhibited significant positive linear correlations with RWC, and the slope did not differ among provenances (Fig. 4a–d). By contrast, Pro exhibited negative linear correlations with RWC only in AH and HN provenances, but with similar slopes (Fig. 4e–h). Furthermore, the recovery of *A*_sat_ exhibited significant positive correlations with SOD in most provenances, and the slope was significantly lower in HN and JX compared with AH (Fig. 5a–d). However, *A*_sat_ exhibited negative correlations with Pro except for GX provenance, but the slopes did not differ (Fig. 5e–h).

## Discussion

**Figure 6:**
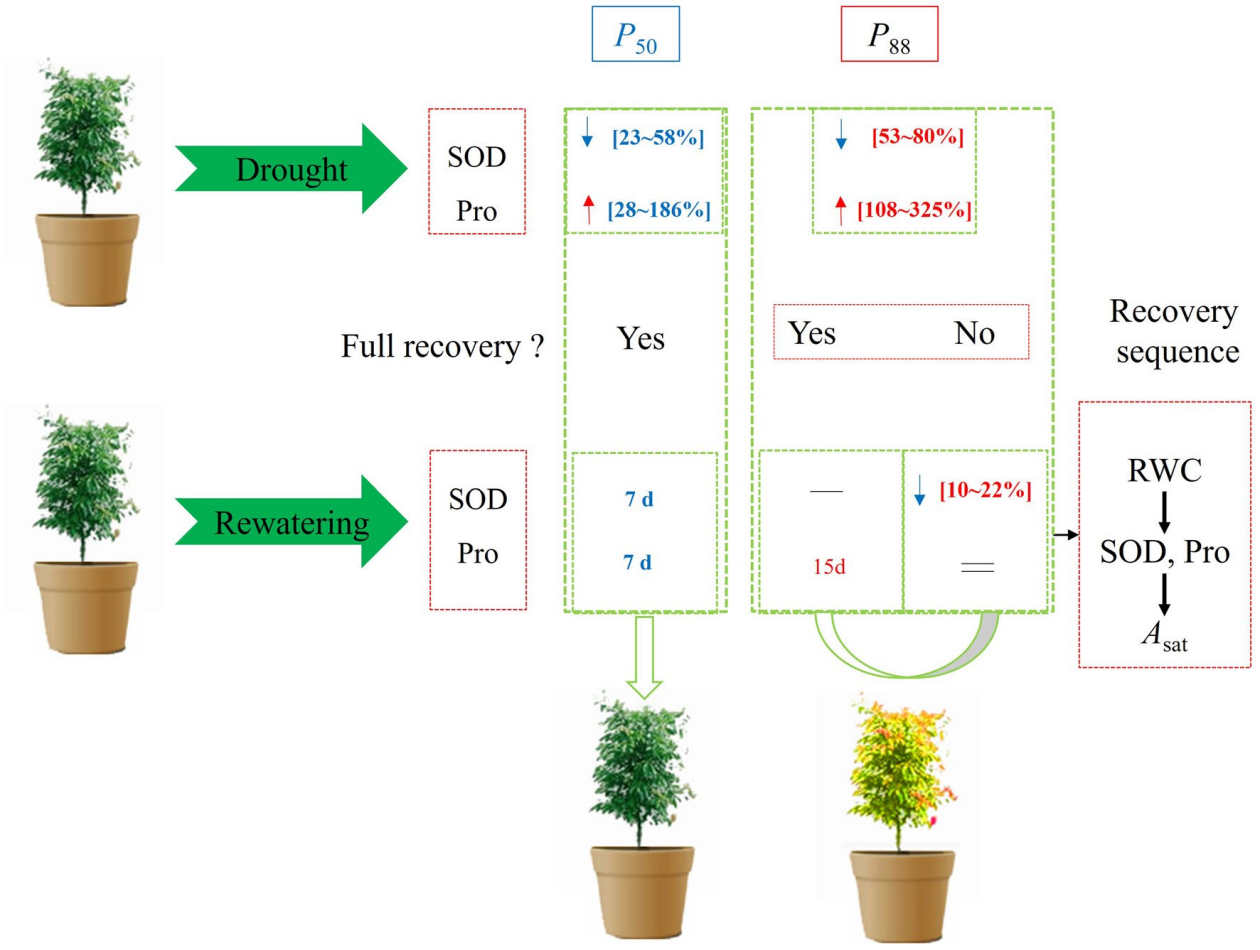
A diagram that shows the biochemical processes during drought and rewatering and the sequence of trait recovery. Drought decreased the SOD activity and increased Pro concentration*,* and the percentage of increase or decrease of *S. superba* under *P*_88_ stress was higher than that under *P*_50_ stress. The recovery pattern differed partially among provenances. In addition, for *P*_50_ recovery, RWC was firstly recovered (3 days), followed by SOD and Pro (7 days), finally driving recovery in *A*_sat_ (≥ 7 days). For *P*_88_ recovery, Pro was recovered in 15 days, while SOD was not recovered in 30 days. “Yes” indicates that it has been returned to the control, and “No” represents it cannot be restored. The blue arrows represent decrease, and the red arrows indicate increase. “–” represents that it cannot be restored and “=” represents that there was no observed value because they were recovered earlier. The number indicates the percentage of increase or decrease compared with the control.

Main findings are synthesized in Fig. 6. Results showed that (a) greater drought intensity (*P*_88_ stress compared to *P*_50_ stress) generated lower SOD activity and increased Pro concentration of *S. superba* seedlings similarly in all provenances, thus leading to slower recovery in seedlings subjected to more severe antecedent drought stress. (b) In contrast, the recovery of some physiological and biochemical indicators (i.e. mainly *A*_sat_ and SOD activity) differed partially among provenances. (c) There were differences in the rate of recovery of processes following rewatering, with the order from RWC, SOD, Pro and finally *A*_sat_. (d) During rewatering, SOD activity and Pro concentration exhibited correlations with RWC, and then affected *A*_sat_ recovery with different patterns among provenances.

### Responses to drought

Drought stress can increase reactive oxygen species (ROS) in plants and cause membrane lipid peroxidation, cell damage and death, because the excessive production of ROS by plant cells under stress is highly reactive and toxic to proteins, lipids, carbohydrates and nucleic acids^42^. Hence, plants often produce a series of antioxidant enzymes (e.g. SOD, POD, CAT) to counteract the damage caused by ROS^17,43^. However, the present study showed that the activity of SOD was decreased significantly with the increasing degree of drought stress (i.e. from *P*_50_ stress to *P*_88_ stress). The earlier study found that gas exchange dropped to zero before *P*_50_-stress^9^, indicating that SOD of *S. superba* was likely dissociated under severe stress and thus it is unlikely to perform the corresponding role^44,45^. In fact, drought responses of SOD are mixed, depending on the degree of drought stress. Despite that our finding was inconsistent with some previous studies under mild to moderate drought stress^46,47^, it agrees with other studies showing that SOD declined as drought was severe^48–50^.

By contrast, Pro concentration was gradually increased with increasing drought stress, suggesting that proline accumulation may allow *S. superba* to scavenge ROS. Along with previous studies, it is shown that proline can effectively maintain the balance of cell osmosis and the integrity of cell membrane, and was regarded as a scavenger of ROS^51–53^. Collectively, above results demonstrate that *S. superba* may have the physiological strategy to cope with severe drought stress, mainly through increasing the ability of osmotic regulation rather than scavenging superoxide anions.

### Responses to rewatering

Greater drought intensity (*P*_88_ stress compared to *P*_50_ stress) led to slower physiological recovery in seedlings, which is similar to previous results that the rate of recovery was slower when the seedlings were exposed to greater drought stress^12,49,54,55^. In line with earlier studies, the SOD activity of droughted seedlings returned to the control status after rewatering from lower degree of drought stress, while it could not recover from a severe drought^19,56^. It shows that rewatering can alleviate the physiological damage to seedlings caused by mild or moderate drought stress, but the enzyme synthesis may be blocked under severe drought, resulting in slow recovery or inability of recovery of SOD activity^55^. Previous evidence has demonstrated that the ability of osmotic regulation declined with increasing degree of drought stress^57–59^, with no recovery when approaching severe stress. However, in this study, it was observed that Pro could recover even after rewatering from *P*_88_ stress, which is usually thought to be lethal stress in earlier studies, suggesting that leaf cell of *S. superba* seedlings has the capacity to regulate osmotic function once the severe drought is released. In addition, SOD and Pro exhibited significant linear correlations with RWC. Increasing RWC after drought can improve plant cell integrity. On one hand, it reactivates the protection capacity of SOD and scavenges excess superoxide anions^47,60,61^. On the other hand, plant cells increase water absorption capacity, thus eliminating the need for additional Pro synthesis for osmotic protection^5,62^. However, the relationship between Pro and RWC was not significant for GX and JX seedlings, mainly due to the lack of recovery in proline concentration after 7 days of rewatering from *P*_88_ stress, even when RWC returned to controlled values. This indicates that GX and JX seedlings had lower capacity to regulate proline after severe drought stress than other provenances. Therefore, intra-specific variation in the capacity of regulating proline after drought should be considered in evaluating drought resilience of *S. superba*.

A clear hierarchical recovery from drought in physiological processes in seedlings of *S. superba* was observed. RWC recovered most rapidly from drought stress, followed by an increase in SOD activity and reduction in Pro concentration, which can promote the recovery of cell membrane function by alleviating cell membrane damage^63,64^. Decreasing RWC is associated with cell membrane disruption^65,66^. Therefore, the increase of RWC can improve the integrity of cell membrane and further help with the recovery of SOD and Pro in this study, thus maintaining the structural stability of plant cell membranes and proteins, and finally restoring photosynthesis^67^. Moreover, the recovery of *A*_sat_ was significantly correlated with SOD and Pro, indicating that the change of *A*_sat_ was affected by both stomatal and non-stomatal factors^68,69^. Nevertheless, these relationships were not significant for GX seedlings mainly due to the slower recovery in *A*_sat_, demonstrating that some other non-stomatal factors such as damage to the photosystem may contribute to this process. For example, the damage to the provenance in GX was found to be more severe under the same degree of drought^70^. In addition to the limitation of photo biochemistry (i.e. *V*_cmax_ and *J*_max_) on *A*_sat_^71,72^, this study further enhances the understanding of biochemical effects by highlighting the role of cell membrane antioxidant enzymes and osmotic regulations. Consequently, partial recovery of photosynthetic capacity was restored after the cell function was recovered.

### Intra-specific differences in response to drought and rewatering

Previous studies have shown that there was a significant correlation between intra-specific drought resistance and leaf ultrastructure^23,73^. In this study, however, leaf ultrastructural traits did not vary among provenances at pre-drought conditions, which is in agreement with other studies^74,75^. The duration of dry down process was relatively short (10–12 days) in this experiment and might have little chance to change the leaf ultrastructure substantially prior to drought and subsequent rewatering, thus it is speculated that leaf ultrastructure should not play roles in the intra-specific difference in recovery of leaf physiology of *S. superba* seedlings. Therefore, leaf ultrastructural traits of *S. superba* seedlings may have little capacity of plasticity to acclimate the climate origin, at least based on the four provenances used in this study. Despite this, further exploring longer term leaf ultrastructure plasticity during the drought and rewatering stages can help us to tears its role apart from physiological and biochemical factors.

We observed that the responses of SOD, *A*_sat_ and Pro showed intra-specific differences and *A*_sat_ exhibited significant correlation with SOD and Pro. The decreased *A*_sat_ was largely coupled with stomatal conductance (*g*_s_)^9^, similar with previously observed results^14,76,77^. In addition, the intra-specific differences in *A*_sat_ may be modified by SOD activity of provenances, reflected by their different positive correlations among provenances. Furthermore, some studies have showed that the large differences in rainfall, temperature and soil can lead to great variability among provenances^78–80^. For example, provenances seem more drought resistant in drier climates, because they can have greater resistance to embolism and metabolic capacity^81^. However, we observed that the responses of SOD, *A*_sat_ and other indicators were not explained by the climate of origin. The soil used was similar among provenances, thus it should not contribute to the intra-specific variation in this study. Therefore, it is difficult to unravel the mechanism driving the intra-specific difference in SOD and other physiological indicators based on the current experimental design and it is beyond the scope of this study. At least, however, the role of SOD on *A*_sat_ recovery was determined. Furthermore, *g*_min_ was higher in HN than other provenances, indicating that leaf water loss after stomatal closure was faster in HN. However, whether this difference is related to other leaf structural traits and how they may be correlated with physiological recovery deserve further studies. Additionally, we suggest that intra-specific differences in physiology and biochemistry in response to drought and following rewatering should be more considered to distinguish plant drought strategies within species under future climate change.

## Conclusions

This study revealed that there were different patterns of physiological and biochemical responses in the four provenances of *S. superba* during drought stress and following rewatering. These differences may provide greater understanding of the diversity of drought strategies of *S. superba* seedlings in subtropical forests of China under future climate change. Drought significantly reduced SOD activity of* S. superba* similarly among provenances, associated with similar leaf anatomical structure. Seedlings from all provenances mainly increased the concentration of osmotic regulatory substances (Pro) to improve cellular and plant water status to cope with drought stress, varying with the extent of changes. The recovery patterns of some physiological and biochemical traits differed partially among provenances, but they were not related to the climate of origin. However, there was a similarly clear progression of process recovery following rewatering among provenances, led by the recovery of RWC, followed by SOD and Pro, and finally *A*_sat_. These traits were highly correlated in some provenances but not in others.

It is worth noting that the experiment was conducted in the general peak growth period (August) for *S. superba*. Therefore, caution should be paid when putting forward this finding to a general pattern, because plants may have different response strategies to drought during different phenological periods and stages of nutritional and reproductive growth. Furthermore, results in this study are from potted seedlings, which need careful caution when extrapolating to mature trees in the field. In summary, the intra-specific differences of *S. superba* seedlings in recovery of physiology and biochemistry following rewatering highlight the need to consider variations within a given tree species coping with future more frequent drought stress.

## Data Availability

The datasets used and/or analysed during the current study are available from the corresponding author on reasonable request.
